# Computer-based alcohol reduction intervention for alcohol-using HIV/HCV co-infected Russian women in clinical care: study protocol for a randomized controlled trial

**DOI:** 10.1186/s13063-021-05079-x

**Published:** 2021-02-17

**Authors:** Ralph J. DiClemente, Jennifer L. Brown, Ariadna Capasso, Natalia Revzina, Jessica M. Sales, Ekaterina Boeva, Lyudmila V. Gutova, Nadia B. Khalezova, Nikolay Belyakov, Vadim Rassokhin

**Affiliations:** 1 School of Global Public Health, New York University, New York, NY USA; 2 Department of Psychology, University of Cincinnati, Cincinnati, OH USA; 3 Addiction Sciences Division, Department of Psychiatry & Behavioural Neuroscience, University of Cincinnati College of Medicine, Cincinnati, OH USA; 4 Center for Addiction Research, University of Cincinnati, Cincinnati, OH USA; 5 Clinical Trials Compliance, Office for Clinical Research, School of Medicine, Emory University, Atlanta, GA USA; 6 Rollins School of Public Health, Emory University, Atlanta, GA USA; 7 First Saint Petersburg State Pavlov Medical University, Saint Petersburg, Russia; 8 Saint Petersburg Pasteur Institute, Saint Petersburg, Russia

**Keywords:** HIV, Hepatitis C virus, HIV/HCV co-infection, Russia, Women, Service integration, Alcohol reduction intervention, Computer-delivered alcohol intervention, Randomized controlled trial

## Abstract

**Background:**

Russia has a high prevalence of human immunodeficiency virus (HIV) infections. In 2018, over one million persons were living with HIV (PLWH); over a third were women. A high proportion of HIV-infected women are co-infected with hepatitis C virus (HCV), and many consume alcohol, which adversely affects HIV and HCV treatment and prognosis. Despite the triple epidemics of alcohol use, HIV and HCV, and the need for interventions to reduce alcohol use among HIV/HCV co-infected women, evidence-based alcohol reduction interventions for this vulnerable population are limited. To address this gap, we developed a clinical trial to evaluate the efficacy of a computer-based intervention to reduce alcohol consumption among HIV/HCV co-infected women in clinical care.

**Methods:**

In this two-arm parallel randomized controlled trial, we propose to evaluate the efficacy of a culturally adapted alcohol reduction intervention delivered via a computer for HIV/HCV co-infected Russian women. The study population consists of women 21–45 years old with confirmed HIV/HCV co-infection who currently use alcohol. Intervention efficacy is assessed by a novel alcohol biomarker, ethyl glucuronide (EtG), and biomarkers of HIV and HCV disease progression. Women are randomized to trial conditions in a 1:1 allocation ratio, using a computer-generated algorithm to develop the assignment sequence and concealment of allocation techniques to minimize assignment bias. Women are randomized to either (1) the computer-based alcohol reduction intervention or (2) the standard-of-care control condition. We will use an intent-to-treat analysis and logistic and linear generalized estimating equations to evaluate intervention efficacy, relative to the standard of care, in enhancing the proportion of women with a laboratory-confirmed negative EtG at each research study visit over the 9-month follow-up period. Additional analyses will evaluate intervention effects on HIV (viral load and CD4+ levels) and HCV markers of disease progression (FibroScan).

**Discussion:**

The proposed trial design and analysis provides an appropriate conceptual and methodological framework to assess the efficacy of the computer-based intervention. We propose to recruit 200 participants.

The intervention, if efficacious, may be an efficient and cost-effective alcohol reduction strategy that is scalable and can be readily disseminated and integrated into clinical care in Russia to reduce women’s alcohol consumption and enhance HIV/HCV prognosis.

**Trial registration:**

ClinicalTrials.gov NCT03362476. Registered on 5 December 2017

## Administrative information

The order of the items has been modified to group similar items (see http://www.equator-network.org/reporting-guidelines/spirit-2013-statement-defining-standard-protocol-items-for-clinical-trials/).

**Table:** 

Title {1}	Computer-Based Alcohol Reduction Intervention for Alcohol-Using HIV/HCV Co-Infected Russian Women in Clinical Care: A Study Protocol for a Randomized Controlled Trial
Trial registration {2a and 2b}.	ClinicalTrials.gov, registration number NCT03362476
Protocol version {3}	Last updated: October 31, 2019
Funding {4}	Research reported in this publication was supported by the National Institute on Alcohol Abuse and Alcoholism of the National Institutes of Health under Award Number R01AA025882 (Multiple Principal Investigators: RJD, JLB) and by a grant 17-54-30014 from the Russian Foundation for Basic Research (Principal Investigator: SP).
Author details {5a}	^1^ School of Global Public Health, New York University, New York, New York, United States ^2^ Department of Psychology, University of Cincinnati; Addiction Sciences Division, Department of Psychiatry & Behavioural Neuroscience, University of Cincinnati College of Medicine; Center for Addiction Research, University of Cincinnati, Cincinnati, Ohio, United States ^3^ Clinical Trials Compliance, Office for Clinical Research, School of Medicine, Emory University ^4^ Rollins School of Public Health, Emory University, Atlanta, Georgia, United States ^5^ First Saint Petersburg State Pavlov Medical University, Saint Petersburg, Russia ^6^ Saint Petersburg Pasteur Institute, Saint Petersburg, Russia
Name and contact information for the trial sponsor {5b}	National Institute on Alcohol Abuse and Alcoholism of the National Institutes of Health, Bethesda, MD, niaaaweb-r@exchange.nih.gov, 301-443-3860
Role of sponsor {5c}	The funding sources had no role in the study design; collection, management, analysis, or interpretation of data; writing of the report; or the decision to submit the report for publication, nor do they have the ultimate authority over any of these activities.

## Introduction

### Background and rationale {6a}

The human immunodeficiency virus (HIV) and hepatitis C virus (HCV) epidemics are interrelated with increasing prevalence in Russia. As of 2018, over one million persons were living with HIV (PLWH) in the Russian Federation (Russia) [1]. Women made up 37% of new HIV diagnoses, almost tripling the 2006 prevalence rate [1].

The severity of the HIV epidemic is compounded by the intersecting HCV epidemic. Eastern Europe has the largest proportion of PLWH with serological evidence of HCV infection (27%) [2]. The prevalence of HIV/HCV co-infection is markedly higher among individuals with a history of injection drug use (PWID). A recent review estimated an HCV prevalence of 69% among PWID compared to 4.6% among PLWH without a history of injection drug use in Russia [3]. Among HIV-infected women in Saint Petersburg, Russia, a study by our group observed that 57.1% were HCV co-infected [4]. Behaviours related to injection drug use, such as needle sharing, have contributed to the rapid spread of HIV/HCV co-infection among PWID globally [5], including in Saint Petersburg, Russia [6–8].

Alcohol use poses a significant and persistent health threat to women living with HIV/HCV co-infection. Despite substantial reductions in alcohol use over the past two decades [9], problem alcohol use is prevalent in Russia and adversely impacts the treatment and prognosis of women living with HIV/HCV by accelerating HIV and HCV progression [10]. A recent meta-analysis estimated that 55% [95% confidence interval (CI) 16–88%] of Russian participants engaged in heavy episodic drinking and 58% (95% CI 49–67%) met diagnostic criteria for alcohol use disorder [11]. A World Health Organization study estimated that among Russian women, 43.7% of those who drank were heavy episodic drinkers compared to 19.9% in Europe as a whole [12]. A recent study among PLWH in Saint Petersburg found that, overall, 45.3% of women reported drinking in the past week. Of those, 21.6% reported at least one heavy episodic drinking episode (4 or more drinks) [13].

Among HIV/HCV co-infected individuals, alcohol use may also lead to various adverse health outcomes. HIV/HCV co-infected individuals develop liver disease earlier and are diagnosed with more severe liver disease than those who are HIV mono-infected [14–16]. Excessive alcohol consumption is a significant contributor to liver-related mortality among persons with HIV/HCV co-infection [17]. The adverse impact of alcohol use may be worse for women, who are more prone to rapid fibrosis progression and cirrhosis than men [18]. In addition to direct health complications, alcohol may heighten health risks to PLWH by interacting with antiretroviral (ARV) therapy. For example, alcohol and ARV interactions may contribute to hepatoxicity and liver disease [14, 16], which may be accelerated by co-morbid HCV infection [19]. Furthermore, a history of alcohol use may impede persons with HIV/HCV co-infection from benefiting from the new generation of HCV treatments, such as direct acting anti-HCV drugs [20]. Current recommendations are for individuals with HIV/HCV co-infection to abstain from alcohol use given that safe drinking levels have not been defined for this population [21]. There is conflicting evidence concerning the health effects of moderate alcohol consumption among HCV mono-infected and HCV/HIV co-infected persons, with some studies finding that even moderate amounts of alcohol may accelerate disease progression, while other studies identify no such association [22, 23].

Despite the need for interventions to reduce alcohol use among HIV/HCV co-infected women, few evidence-based interventions are available [10, 24]. A recent review of interventions to reduce alcohol consumption among PLWH identified limited evidence that interventions reduced binge drinking frequency, alcohol use disorders, or alcohol dependence symptoms [10]. Furthermore, most of the interventions did not target alcohol use exclusively, but addressed other high-risk behaviours (e.g. reducing sexual risk behaviours) or focused on substance use more broadly. In addition, the majority of interventions were conducted in the USA; to our knowledge, none was implemented and evaluated in Russia [10].

In this two-arm parallel randomized controlled trial (RCT), we propose to evaluate the efficacy of a brief, culturally adapted, computer-delivered alcohol reduction intervention for Russian women with HIV/HCV co-infection. The intervention includes modules utilizing cognitive behavioural and motivational enhancement principles to reduce alcohol consumption. Brief interventions based on cognitive behavioural therapy that includes personalized feedback about alcohol use, the establishment of personal drinking goals, and tracking of goal progress coupled with behavioural strategies to reduce alcohol use are efficacious [25, 26].

The proposed intervention will be brief and delivered via a computer platform. Computer-delivered alcohol reduction interventions effectively lower problematic alcohol use, albeit with small effect sizes [27, 28] and may be more cost-effective and feasible to integrate into clinical care settings than existing interventions delivered by experienced, trained clinical providers. A mounting body of evidence points to the promise of integrating treatment for alcohol use within comprehensive HIV-care services [29]. If efficacious, our intervention may be a scalable and cost-effective strategy to reduce alcohol use among women with HIV/HCV co-infection within the framework of comprehensive HIV care, in line with current strategies to strengthen HIV-care delivery.

## Objectives {7}

The study objectives are:

1. To evaluate the efficacy of a computer-based alcohol reduction intervention condition, relative to the standard-of-care condition, in enhancing the proportion of HIV/HCV co-infected women detected with laboratory-confirmed negative alcohol biomarker over a 9-month follow-up period

2. To evaluate the efficacy of a computer-based alcohol reduction intervention condition, relative to the standard-of-care condition, in reducing HIV/HCV co-infected women’s HIV and HCV biomarkers of disease progression over a 9-month follow-up period

## Trial design {8}

The proposed study is a parallel two-arm RCT to evaluate the efficacy of a culturally adapted computer-based alcohol reduction intervention for HIV/HCV co-infected Russian women in clinical care.

Following receipt of informed consent and enrolment in the study, 200 women are randomized to one of two conditions following a 1:1 allocation ratio: (1) standard of care plus the adapted computer-based intervention condition (*n* = 100) or (2) the standard-of-care control condition alone (*n* = 100).

**Fig. 1:**
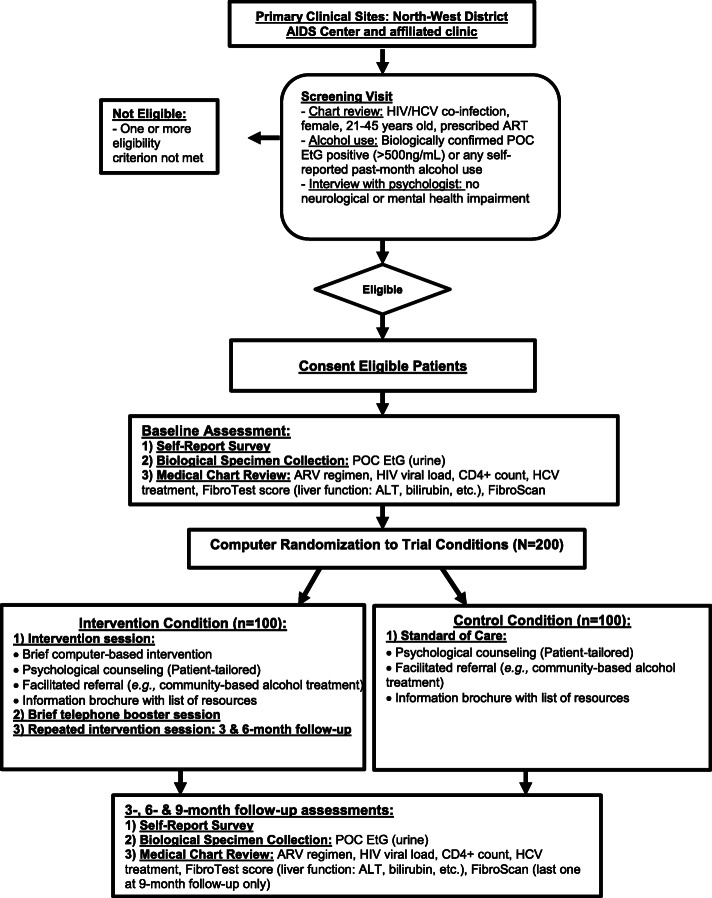
Trial flow diagram

Data are collected at baseline, before randomization, and at 3-, 6-, and 9-month follow-up during scheduled research visits via self-report surveys. In addition, biological markers are collected at baseline and at each follow-up assessment to obtain objective and quantifiable measures of alcohol use and HIV and HCV disease status. The proposed trial design provides an appropriate conceptual and methodological framework to determine the intervention’s efficacy in reducing alcohol use and HIV and HCV disease progression (see Fig. 1).

## Methods: participants, interventions, and outcomes

### Study setting {9}

The intervention is implemented in HIV clinical care facilities in Saint Petersburg, Russia. Saint Petersburg is the second largest city in Russia, with a population of approximately 5 million. It has a high concentration of PWID, a critical risk behaviour associated with HIV/HCV acquisition [6]. An estimated 40,000 PLWH live in Saint Petersburg, with about 2000 new cases diagnosed annually [30]. There is a high prevalence of HCV infection among Russian PLWH [4]. The current study includes women seeking HIV care at two clinical care sites. Both sites offer comprehensive HIV care, including mental health care, for HIV-infected women.

### Eligibility criteria {10}

Eligible participants must (1) be women ages 21–45 years, (2) have chart-documented HIV and HCV infection, (3) be currently prescribed ARV therapy, and (4) have a positive point-of-care ethyl glucuronide (EtG) or self-report alcohol use in the past 30 days. Women who are medically, cognitively, or psychologically incapable of study participation, as assessed by a research clinician, are excluded, as those who do not provide written informed consent to participate.

### Who will take informed consent? {26a}

A research staff member explains the study, reviews the consent form with participants, and answers any questions before women provide written consent to participate in the study. All eligible participants must complete consent procedures before enrolment and randomization. Measures are being implemented to protect study participants. Study staff are instructed to highlight confidentiality and assure participants that their responses would not be shared with clinic staff or adversely affect service provision.

#### Compensation

Participants receive a 1000-ruble (~ 16 United States Dollars (USD)) valued gift card at baseline and at the 9-month follow-up visit and a 500-ruble (~ 8 USD) valued gift card for each completed intermediary assessments (3- and 6-month research visits).

### Additional consent provisions for collection and use of participant data and biological specimens {26b}

The consent form explains to participants that de-identified data will be part of a general dataset that may be shared with other researchers. In terms of biological specimens, the consent form explains that if participants choose to join the study, they will be donating their samples and study information. If they withdraw from the study, data and samples collected may still be used for this study. Participants are informed that study records can be opened by court order and may be provided in response to a subpoena or a request for the production of documents. Further study records may be shared with regulatory authorities, such as the IRB, government agencies, and study funders.

## Interventions

### Explanation for the choice of comparators {6b}

This trial compares the efficacy of standard clinician-delivered alcohol reduction counselling to a computer-delivered alcohol use reduction intervention. We hypothesize that the computer-delivered intervention may be less costly, less demanding on human resources, and more feasible to integrate into clinical care than the current standard of care, with comparable results.

### Intervention description {11a}

#### Intervention condition

Women in the intervention condition receive a brief computer-based intervention to reduce alcohol use guided by cognitive behavioural principles. The computerized intervention includes modules to (a) provide an overview of the intervention and information regarding the impact of alcohol use on disease progression, (b) assess current levels of drinking and provide feedback on alcohol use, (c) set alcohol reduction goals and enhance motivation to modify drinking behaviours, (d) address challenges associated with achieving drinking goals, and (e) provide strategies to reduce alcohol use. The computerized intervention was adapted to be culturally appropriate and incorporates vignettes to illustrate intervention content. After each computer-based session, a study clinician meets with participants to establish drinking goals and discuss strategies to achieve these goals during a brief one-on-one motivational enhancement session. Participants randomized to the intervention condition also continue to receive the standard of care for alcohol use provided in the clinic.

#### Standard-of-care condition

Women receiving treatment for HIV/HCV at the two clinical care sites are routinely asked about their alcohol and other substance use and counselled to avoid alcohol and other substances by clinical staff. When the treating clinician deems it appropriate, women are referred to community-based care for substance use treatment. In addition, all women receive an educational brochure with information describing the adverse effects of alcohol use for persons with HIV/HCV co-infection and a list of community-based resources to seek further help.

### Criteria for discontinuing or modifying allocated interventions {11b}

A staff psychiatrist assesses women’s mental health as part of routine care and as requested by a treating clinician and refers women diagnosed with alcohol dependence to substance use treatment. The psychiatrist may recommend discontinuing participation in the trial if a participant is deemed psychologically incapable of continued participation.

### Strategies to improve adherence to interventions {11c}

The intervention consists of taking a brief computer-based programme; therefore, the key strategy to promote adherence is the outreach efforts to invite women back to the clinic to complete the first and follow-up sessions. To improve adherence, research staff will call participants to schedule visits, using the contact information on file at the clinical sites.

### Relevant concomitant care permitted or prohibited during the trial {11d}

All routine treatment procedures for women in care for HIV and HCV, as deemed appropriate by the patient’s treating clinicians, are permitted during the trial.

### Provisions for post-trial care {30}

Trial participation does not present more than minimal risk to subjects beyond those encountered as part of routine care. Therefore, no special provisions for post-trial care were included.

### Outcomes {12}

Biomarkers (EtG and phosphatidylethanol (PEth)) are used to assess the efficacy of the intervention on alcohol use patterns and two associated health outcomes, HIV and HCV, as described below.

The primary outcome measure used to evaluate the efficacy of the adapted computer-based alcohol reduction intervention condition, relative to the standard-of-care condition, will be the proportion of women who test EtG negative at each of the follow-up assessments over 9 months.

HIV disease progression is evaluated based on changes in HIV viral load and CD4+ count over the 9-month follow-up period in the intervention arm relative to the control arm. HCV disease progression is assessed using two non-invasive biomarkers collected routinely as part of ongoing HCV care. The first is FibroTest which uses six different biomarkers (α2-macroglobulin, haptoglobin, γ-glutamyl transpeptidase, apolipoprotein A1, alanine transaminase, and total bilirubin) to generate a score that is correlated with the degree of liver damage [31]. The second is FibroScan, a specialized ultrasound that measures the degree of fibrosis and steatosis in the liver, providing objective and quantifiable measures of HCV-associated liver damage [32]. The procedure is painless and performed at point-of-care.

### Participant timeline {13}

**Fig. 2:**
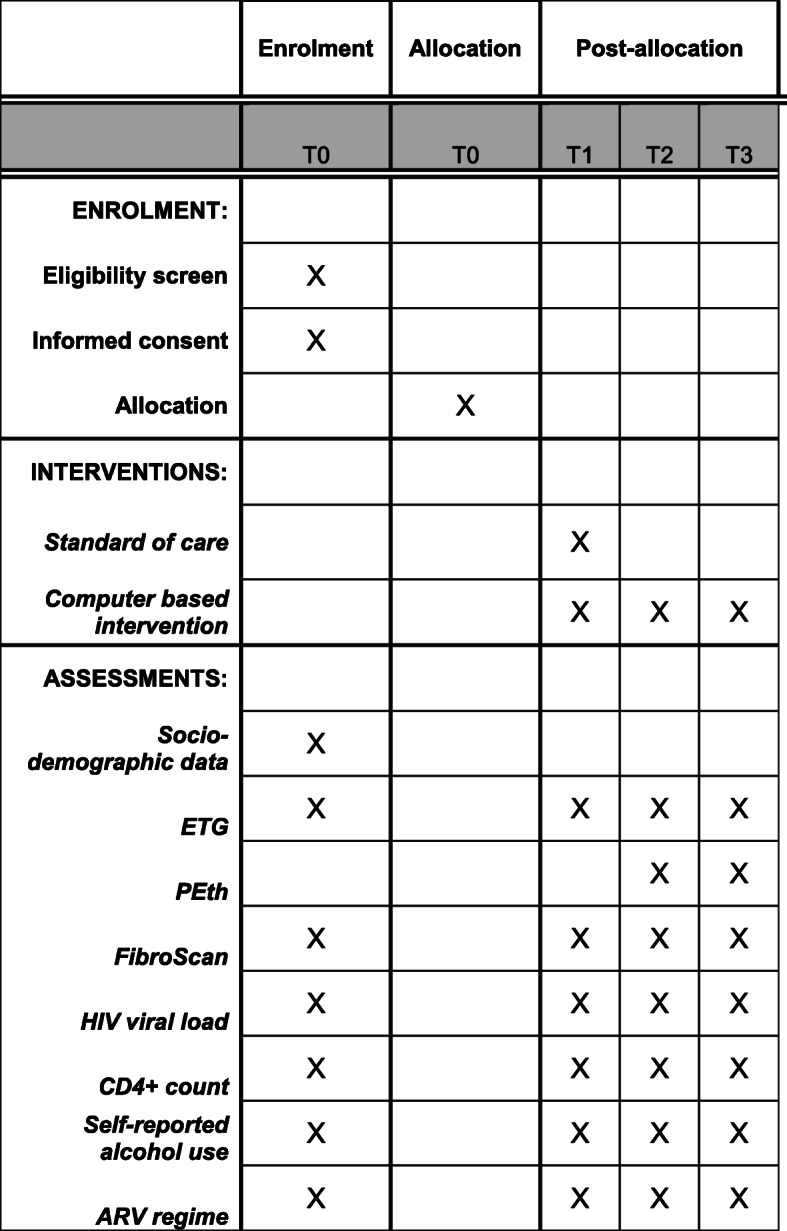
Standard Protocol Items: Recommendations for Interventional Trials (SPIRIT) schedule of enrolment, interventions, and assessments

The participant timeline is presented in Fig. 2.

### Sample size {14}

The sample size of 200 women (1:1 allocation ratio) was arrived at based on a power analysis. The most conservative hypothesis compares differences between trial conditions in the proportion of participants detected with a negative EtG test over the 9-month follow-up. EtG was selected for the power analysis because it is a sensitive biological test of alcohol use and has a broad alcohol detection window (5 days). The power calculations for the negative EtG test were based on our previous pilot study in which 30% of HIV/HCV-infected Russian women were identified with a negative alcohol use biomarker [33]. Our study is powered to detect a small effect (delta = − 0.2; odds ratio = 0.3) in the number of women testing EtG negative under a repeated measures logistic regression analysis framework, assuming an intra-cluster correlation of 0.10 and 10% loss-to-follow-up. Analyses were conducted in Stata 15.1 (Stata Corporation LP, College Station, TX).

### Recruitment {15}

Recruitment is conducted at two affiliated clinical sites that provide comprehensive HIV care. The study coordinator in Russia, a medical doctor with specialization in infectious diseases, reviews medical records to assess potential eligibility. A clinician at each clinic then invites participants to the study and schedules a follow-up visit with a research staff member.

## Assignment of interventions: allocation

### Sequence generation {16a}

A computer algorithm is used to generate a random allocation sequence prior to the study initiation.

### Concealment mechanism {16b}

We use concealment of allocation techniques to promote unbiased randomization [34]. Predetermined allocations are sequentially numbered.

### Implementation {16c}

The random allocation sequence is generated in the USA and shared with Russian staff to implement. Only the Russian-based study coordinator has the list of allocations generated in the USA and informs other researchers of the allocation following participant screening and enrolment.

## Assignment of interventions: blinding

### Who will be blinded {17a}

Because the intervention entails completing a computer-based programme, neither providers nor participants are blinded to the intervention assignment; however, the statisticians conducting the efficacy analyses will be blinded to allocation. Research members in Russia will assign a code to each of the two arms, which will be undisclosed to US-based data analysts.

### Procedure for unblinding if needed {17b}

The design is an open label, so unblinding will not occur.

## Data collection and management

### Plans for assessment and collection of outcomes {18a}

Data for efficacy assessment is collected at baseline and 3, 6, and 9 months post-intervention initiation using multiple data-capture modalities. Participants complete a brief self-report survey assessing basic demographics and history of alcohol and other substance use. In addition to basic demographics, such as age, education, and income, the brief survey includes validated self-report measures of alcohol use (e.g. alcohol use frequency and quantity, and heavy episodic drinking) [35]. The trained attending clinicians, one at each clinic, collect two biomarkers: (1) a urine sample from each woman at each study visit for EtG analysis, which assesses recent alcohol use, and (2) a dried blood spot at the 9-month follow-up for PEth analysis, which measures alcohol use over the past 21 days. A research staff member abstracts via medical record review the following data: (a) length of HIV and HCV infection, (b) HIV viral load, (c) cluster of differentiation 4 cells (CD4+) count, (d) FibroTest score (based on liver function biomarkers, such as apolipoprotein A1, alanine transaminase, and total bilirubin, etc.), (e) prescribed ARV regime, and (f) HCV treatment. Research staff consisted of clinicians experienced in the care of PLWH who received training on implementation of study procedures, including on EtG administration at point-of-care.

EtG is an ethanol metabolite widely used to detect any alcohol consumption in the past 24 h [36]. When measured in urine at point-of-care, it is estimated to have a sensitivity of 100% and a specificity of 97% [37]. PEth is a well-established marker of past 3-week alcohol use [38], including among PLWH [39, 40]. Among PLWH, the sensitivity for any detectable PEth has been estimated at 88% and the specificity at 89% [40]. Collecting three different alcohol use measures—EtG, PEth, and self-reported use—will allow us to triage the information and assess measurement errors and reporting biases. FibroTest is a scoring system based on biochemical markers to evaluate the stage of fibrosis among patients with HCV and is considered a safe, non-invasive alternative to liver biopsy [41, 42]. FibroTest has satisfactory predictive validity of fibrosis staging, with areas under the curve ranging from 73 to 80% depending on liver disease progression compared to biopsy results [42].

### Plans to promote participant retention and complete follow-up {18b}

Based on a previous trial in this population [43] and on pilot work during adaptation of the computerized intervention, we expect loss-to-follow-up to be minimal and not to exceed 10%. Factors to improve intervention adherence and prevent loss-to-follow-up over the 9-month follow-up period are that all research personnel and clinical staff are highly qualified and trained in working with PLWH, that most patients have a trusting relationship with study clinicians, and that all research staff is from Saint Petersburg. In addition, staff will call participants to schedule subsequent research visits. Participants’ contact information is on file at the clinical care sites.

### Data management {19}

In accordance with the National Institutes of Health (NIH) requirements, the clinical trial established a data and safety monitoring plan to ensure participants’ privacy and confidentiality during the trial and as part of data collection and storage. The plan is overseen by a four-member committee composed of the principal investigators and a study coordinator in Russia.

The data safety and monitoring plan stipulates that all records pertaining to the study and all original and electronic data files are to be securely stored off-site, in a locked metal file cabinet, accessible only to one of the Russian-based principal investigators. This is also true for completed and signed consent forms. To ensure participants’ safety and the data’s validity and integrity, only staff with extensive experience in the area of HIV/HCV treatment will be hired. All research staff will receive training on data collection, management and storage procedures (including of survey data and biomarkers), maintaining confidentiality, and research ethics. On an ongoing basis, the Russian-based study coordinator will review the data to ensure data quality, including checking the range of values and identifying any inconsistencies in the data. No names or other identifying information appears on data documents or in data files. Pen and pencil surveys will also contain no identifiers. Only designated staff will have access to the data at each site. Presentations and publications will not disclose the names of the clinical sites where the research was implemented. When the final dataset is made publicly available, data will not contain identifying information.

The data safety and monitoring plan stipulates the following data management procedures, as described in this protocol: data entry is the responsibility of the study coordinator, who enters all data onto an Excel worksheet. The Excel worksheet includes screening information but not randomization information, which is kept in a separate password-protected file. Research personnel at two sites in Russia are in charge of screening procedures and administering the paper-based surveys and of collecting the biomarkers for analysis after randomization. The study coordinator is responsible for assigning allocations based on trial condition assignments generated in the USA. All surveys and biomarkers are marked with a trial identification number, assigned by the study coordinator, to ensure anonymity. One person at each study site is responsible for personally delivering the surveys and the biomarkers to the study coordinator. The study coordinator extracts information from the paper-based surveys, from medical records, and from lab reports to populate the database. The coding used on the general dataset for data entry is based on the survey: each survey item includes numeric codes, and these are used for data entry. The de-identified general database will be password-protected and shared periodically with researchers in the USA for a range of data value checks.

### Confidentiality {27}

All data collected during the study will be kept strictly confidential and only accessed by research staff, unless requested by the research sites’ IRB members or by the sponsor. Participants are assigned a trial identification number. Only the study coordinator has the list linking identifying information with the identification number; this list is password-protected and stored on her personal computer, also password-protected. The de-identified dataset will be available to the rest of the research staff, and after analyses and publication of primary findings will be made available to other researchers upon request.

### Plans for collection, laboratory evaluation, and storage of biological specimens for genetic or molecular analysis in this trial/future use {33}

At baseline and each follow-up assessment, women provide a urine sample, which is analysed for EtG at the North-West District AIDS Center. The limit of detection is > 500 ng/mL. In addition, at the 9-month follow-up, clinicians collect dried blood spot samples, which will be analysed for PEth using US Drug Testing Laboratory standardized procedures (USDTL, Des Plaines, IL). The PEth samples are analysed at the [redacted] in Irkutsk, Russia. The limit of detection is 8 ng/mL; any result above this threshold is considered positive for alcohol consumption within the last 21 days. Urine samples will be disposed of immediately following analysis at point-of-care. The dried blood spot cards will be labelled with a participant number to protect the participants’ identity and stored at − 30 °C at the main clinical site until shipped to Irkutsk for processing. These biological specimens will be destroyed after processing.

## Statistical methods

### Statistical methods for primary and secondary outcomes {20a}

We will use an intent-to-treat analysis and logistic and linear generalized estimating equations to evaluate intervention efficacy, relative to the standard of care, in enhancing the proportion of women with a laboratory-confirmed negative EtG over the follow-up period, thus assessing whether there was a significant reduction in alcohol use in the intervention group, compared to the control group. Given that there is no consensus regarding safe levels of alcohol use among HIV/HCV-infected individuals [44, 45] and current recommendations are to abstain from alcohol use [46], a key outcome is the proportion of women who test EtG negative (< 500 ng/mL) over the 9-month follow-up in each trial condition. To triage results with self-reported patterns of alcohol use patterns and EtG, we will also use a novel alcohol use biomarker, PEth collected once at follow-up. We will conduct multilevel regression analyses to account for clustering effects at the two clinical sites, and between- and within-subject variation. Following the conventions in most trials, we will model treatment differences as a fixed-effect and use random effects for modelling the clustering effects of cohort and repeated measures data.

We will utilize a similar analysis to assess whether the intervention resulted in differences in liver disease progression by evaluating changes in indirect serum markers of liver function based on mean FibroTest scores and direct biomarker of disease progression, FibroScan, and whether the intervention affected HIV disease progression by assessing changes in mean viral load and CD4+ levels between the two conditions.

## Oversight and monitoring

### Composition of the coordinating centre and trial steering committee {5d}

A five-member steering committee was formed, composed of the principal investigators in the USA (RJD, JLB) and in Russia (NB, VR) and the Russian-based study coordinator (EB). The committee’s role is to assess progress and ensure quality and compliance with recruitment and study procedures. The committee meets virtually monthly to evaluate project progress, review modifications, and monitor compliance with guidelines from the relevant institutional ethics committees and The Office for Human Research Protections of the US Department of Health and Human Services. VR supervises the trial operations in Russia. EB is the project coordinator and oversees all aspects of screening, recruitment, consent, and study protocol implementation at the two study sites. EB and LVG are in charge of the identification, screening of potential participants, and recruitment. NBK, a psychiatrist with expertise in treating patients with chronic infectious diseases, interviews and administers the survey to participants. EB and LVG are in charge of collecting the biomarkers. The Russian team (NB, VR, EB, LVG, and NKB) hold coordination meetings weekly.

### Composition of the data monitoring committee, its role and reporting structure {21a}

The data monitoring committee is composed of EB, JLB, and AC. The role of the committee is to conduct quarterly data checks to identify potential errors. In addition, the committee routinely assesses if proper procedures are in place to ensure that data remain confidential and that there are no data breaches. If any problems are identified, the data monitoring committee informs members of the trial steering committee, who, depending on the type of problem, file a report to the respective IRBs and/or the sponsor. The committee is independent of the sponsor and none of the members has competing interests to declare.

### Adverse event reporting and harms {22}

A system for monitoring and reporting adverse events was established. All research team members were trained on recognizing and documenting any unusual events or circumstances that may occur during data collection. Researchers will record their observations on comment cards that will be reviewed by the study coordinator. Should a serious adverse event occur, the study coordinator will immediately inform the principal investigators who, in turn, will immediately notify New York University’s Institutional Review Board (IRB). If an adverse event appears to be research-related, it will be reported to the Office for Human Research Protections of the US Department of Health and Human Services and the funding institution project officer, along with summaries of discussions concerning the event. The funding institution project officer will be informed of any IRB action taken concerning any adverse event. Research staff will be monitored closely by the principal investigators and the study coordinator. Staff deficits will be addressed via additional training, close monitoring for proficiency, and if not adhering to established protocols and procedures, will be terminated.

### Frequency and plans for auditing trial conduct {23}

The principal investigators will conduct semi-annual field visits to assess that procedures are being implemented as planned at all sites. These audits are independent of the sponsor and will entail observation of data collection efforts. The trial steering committee meets monthly to assess progress. Any issues identified during semi-annual field visits or during weekly meetings of the Russian-based field team will be immediately reported to the trial steering committee for a full investigation. In addition, members of the data monitoring committee will meet every 6 months to conduct data checks. Ad hoc emergency meetings of the committee will be called if a data breach or data errors are identified.

### Plans for communicating important protocol amendments to relevant parties (e.g. trial participants, ethical committees) {25}

Changes to the proposed protocol will require a re-submission to the IRB of both institutions and updates to the trial registry. If changes to the protocol are different from what was explain to participants during consent, participants will be duly informed of the modifications and re-consented by research staff before continuing in the study.

## Dissemination plans {31a}

Trial results will be shared in peer-reviewed, open-access publications and at national and international research and policy conferences. The final data set will be available through an open-access data repository.

## Discussion

If efficacious in reducing alcohol use, this brief computer-delivered intervention for alcohol-using HIV/HCV co-infected women in Russia would have several advantages. First, the computer-based platform affords participants greater confidentiality, thus encouraging participants to set realistic goals without perceiving pressure from healthcare providers. The digital platform offers greater flexibility of administration, for example, participants can go through the programme at their own pace. Importantly, the participants’ ability to replay segments of the intervention as needed may reinforce risk reduction by enhancing learning and alcohol reduction messages.

The proposed intervention is novel in several ways. First, it is delivered through a computer-based platform. Computer-delivered alcohol reduction interventions effectively reduce problematic alcohol use, albeit with small effect sizes [27, 28], and may be more cost-effective than existing interventions delivered by experienced clinical providers. A computer-based platform offering a brief intervention is less labour- and cost-intensive than traditional interventions and has the potential to be more feasible for integration into the clinical care setting. Third, the study collects two alcohol use biomarkers to assess intervention efficacy, rather than relying on self-report, which is prone to reporting biases. Data from a previous study with this population indicated that alcohol use was often underreported [33]. It will also utilize objective markers of liver function and HIV and HCV disease progression.

As the HIV and HCV epidemics continue to grow, increasing the number of people in care, the computerized intervention programme may allow for more patients to receive critical messages related to alcohol use while reducing the burden on trained healthcare providers in an already heavily demanding clinical care setting. A computer-based intervention may be as or more efficacious (the objective of this trial is to assess efficacy relative to the standard of care) than other service delivery modalities but less labour-intensive and less costly to implement and sustain. Because of the ubiquity and affordability of Wi-Fi and portable technology in clinical care settings in Russia and the limited demand of the intervention on healthcare providers’ time, this computer-based intervention may be a feasible and cost-effective option for scale-up and dissemination to other HIV centres in Russia, and even to different clinical settings where alcohol use may be problematic, such as at obstetrician-gynaecologist clinics and maternity hospitals. If efficacious, the intervention may be a feasible and cost-effective way to reach women with problematic drinking patterns at point-of-care and to integrate an alcohol use intervention into comprehensive HIV-delivery services.

## Trial status

All women have completed the baseline assessment and we are currently working on scheduling women for follow-up assessments. The protocol is version number 6, dated 29 October 2019. Recruitment began in January 2018 and was completed in October 2019. The protocol was submitted after the end of recruitment but before the end of the last patient visit. No significant modifications were made to the protocol following the end of recruitment.

## Abbreviations

ARV: Antiretroviral
CI: Confidence interval
CD4+: Cluster of differentiation 4 cells
EtG: Ethyl glucuronide
HCV: Hepatitis C virus
HHS: US Department of Health and Human Services
HIV: Human immunodeficiency syndrome
ICPSR: Inter-University Consortium for Political and Social Research
IRB: Institutional review board
NIH: National Institutes of Health
NYU: New York University
OHRP: Office for Human Research Protections
PEth: Phosphatidylethanol
PLWH: Person living with HIV/AIDS
PWID: Person who injects drugs
USD: United States Dollars
USDTL: US Drug Testing Laboratory standardized procedures
